# Genome-wide methylation, transcriptome and characteristic metabolites reveal the balance between diosgenin and brassinosteroids in *Dioscorea zingiberensis*

**DOI:** 10.1093/hr/uhae056

**Published:** 2024-02-23

**Authors:** Zihao Li, Yi Li, Luyu Geng, Jiachen Wang, Yidan Ouyang, Jiaru Li

**Affiliations:** State Key Laboratory of Hybrid Rice, College Life Sciences, Wuhan University, Wuhan 430072, China; State Key Laboratory of Hybrid Rice, College Life Sciences, Wuhan University, Wuhan 430072, China; State Key Laboratory of Hybrid Rice, College Life Sciences, Wuhan University, Wuhan 430072, China; State Key Laboratory of Hybrid Rice, College Life Sciences, Wuhan University, Wuhan 430072, China; National Key Laboratory of Crop Genetic Improvement and National Centre of Plant Gene Research (Wuhan), Hubei Hongshan Laboratory, Huazhong Agricultural University, Wuhan 430070, China; State Key Laboratory of Hybrid Rice, College Life Sciences, Wuhan University, Wuhan 430072, China

## Abstract

Diosgenin (DG) is a bioactive metabolite isolated from *Dioscorea* species, renowned for its medicinal properties. Brassinosteroids (BRs) are a class of crucial plant steroidal hormones. Cholesterol and campesterol are important intermediates of DG and BR biosynthesis, respectively. DG and BRs are structurally similar components; however, the regulatory network and metabolic interplays have not been fully elucidated. In an effort to decode these complex networks, we conducted a comprehensive study integrating genome-wide methylation, transcriptome and characteristic metabolite data from *Dioscorea zingiberensis*. Leveraging these data, we were able to construct a comprehensive regulatory network linking DG and BRs. Mass spectrometry results enabled us to clarify the alterations in cholesterol, campesterol, diosgenin, and castasterone (one of the major active BRs). The DG content decreased by 27.72% at 6 h after brassinolide treatment, whereas the content increased by 85.34% at 6 h after brassinazole treatment. Moreover, we pinpointed DG/BR-related genes, such as *CAS*s, *CYP90*s, and *B3-ARF*s, implicated in the metabolic pathways of DG and BRs. Moreover, *CAS*s and *CYP90*s exhibit hypomethylation, which is closely related to their high transcription. These findings provide robust evidence for the homeostasis between DG and BRs. In conclusion, our research revealed the existence of a balance between DG and BRs in *D. zingiberensis*. Furthermore, our work not only provides new insights into the relationship between the two pathways but also offers a fresh perspective on the functions of secondary metabolites.

## Introduction

Diosgenin (DG), a spirosteroid found in *Dioscorea* species, has served as an ideal raw material for synthesizing steroid hormone drugs for more than 70 years due to its simplicity and economy, particularly since Marker and his colleagues successfully synthesized progesterone from DG as a starting material in 1943 [1, 2]. DG has been effectively utilized in numerous commercial applications across the food, cosmetic, agricultural, and pharmaceutical fields due to its diverse physicochemical and biological properties [3–6]. It is regarded as one of the top 10 sources of steroids and is the most commonly accepted plant-derived drug [7–9]. Most *Dioscorea* species contain DG in their rhizomes, with ~30 species containing a DG content of >1% by dry weight. The highest recorded content was found in *Dioscorea zingiberensis* C. H. Wright, an endemic species in China, in which the DG content in the rhizomes of a single plant reached 16.15%. *Dioscorea zingiberensis* is currently the world’s most favorable source plant for steroidal hormone drugs [10, 11].

Brassinosteroids (BRs) constitute the sixth hormone class discovered in plants and are polyhydroxysteroid hormones [12]. BRs assume critical functions in plant development and growth, enabling control of processes such as cell elongation, cell division, photomorphogenesis, xylem differentiation, reproduction, and abiotic and biotic stress responses [13–16]. To date, brassinolide has been studied for more than 50 years and has become an essential phytohormone for the growth and development of plants. Various studies have underscored the importance of BRs in plant secondary metabolism and environmental stress adaptation. For instance, exogenous 24-epibrassinolide treatment significantly enhanced the leaf chlorophyll content in cucumber [17] and the production of secondary metabolites. Under nickel stress, 24-epibrassinolide supplementation boosted the capacity of tomato plants to synthesize metabolites [18].

**Figure 1:**
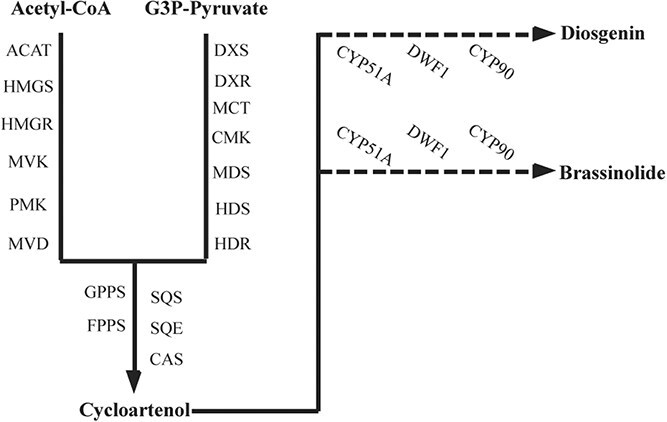
The common parts of biosynthetic pathways of DG and BRs: acetyl Co-A acetyltransferase (ACAT), 3-hydroxy-3-methylglutaryl-CoA synthase (HMGS), 3-hydroxy-3-methylglutaryl-CoA reductase (HMGR), mevalonate kinase (MVK), phosphomevalonate kinase (PMK), mevalonate-5-diphosphate decarboxylase (MVD), 1-deoxy-d-xylulose-5-phosphate synthase (DXS), 1-deoxy-d-xylulose-5-phosphate reductoisomerase (DXR), 2-C-methyl-d-erythritol 4-phosphate cytidylyltransferase (MCT), 4-diphosphocytidyl-2-C-methyl-d-erythritol kinase (CMK), 2-C-methyl-d-erythritol 2,4-cyclodiphosphate synthase (MDS), 4-hydroxy-3-methylbut-2-enyl pyrophosphate synthase (HDS), 4-hydroxy-3-methylbut-2-enyl pyrophosphate reductase (HDR), geranyl pyrophosphate synthase (GPPS), farnesyl pyrophosphate synthase (FPPS), squalene synthase (SQS), squalene epoxidase (SQE), cycloartenol synthase (CAS).

DG and BRs can be classified into spirostanol saponins and cholestanol saponins based on their chemical structure. DG is a type of spirostanol saponin, while BRs are a type of sterol produced from cholestanol [19–22]. Squalene is produced via the methylerythritol phosphate (MEP) and mevalonate (MVA) pathways from acetyl coenzyme A and G3P-pyruvate [21], which results in the formation of cycloartenol. Enzymes such as DWARF1 (DWF1),Cytochrome P450 51A (CYP51A), C-14 reductase (C14-R), sterol 5(6) desaturase (C5-SD), Cytochrome P450 90s (CYP90s), and other CYP450s are involved in subsequent modifications during the biosynthesis of DG. Similarly, BRs are also generated from cycloartenol as a precursor, sharing the same enzymes with DG biosynthesis, such as CYP51A, DWF1, and CYP90s [23–26]. Therefore, DG and BRs share common upstream metabolic pathways and a common synthetic precursor (Fig. 1). However, the mechanism that maintains the synthesis equilibrium of these two substances is still unknown.

BRs are present in most plants, whereas DG is found mainly in plants such as *Dioscorea* species; thus, *Dioscorea* species contain not only BRs but also DG. Castasterone was found to be one of the key BRs within *Dioscorea* species [27]. Due to the species-specific nature of plant secondary metabolites, there are numerous restrictions involved in conducting DG studies with conventional model plants. Undoubtedly, *D. zingiberensis* is the optimal species for conducting DG biosynthesis studies in relation to all DG resource plants. Therefore, it is also optimal to study the relationship between DG and BRs in *D. zingiberensis*. With the successful sequencing of the *D. zingiberensis* genome [28, 29], we have a solid foundation for understanding the interplay between the biosynthesis of DG and BRs, which is a compelling research direction yet to be fully explored in *D. zingiberensis*. By analyzing the association between the two compounds, we can attain the objective of augmenting the amount of DG.

In addition, epigenetic mechanisms that guide plant responses to external stresses are some of the most significant discoveries in recent times. Of these, DNA methylation, one of the earliest-discovered regulatory mechanisms, has been extensively studied in epigenetics. In higher plants, roughly 20–30% of cytosines are methylated, with the level of DNA methylation varying considerably across different plant tissues under different conditions [30, 31]. For instance, soybean nodule development has been suggested to be associated with DNA methylation [32]. Plants can adjust to biotic and abiotic stress during their growth and development [33, 34] by controlling the methylation equilibrium of their genomic DNA. Thus, the metabolism of DG and BRs is closely linked to DNA methylation.

In this study we first determined the balance between DG and BRs with respect to brassinolide (BL) and brassinazole (BRZ) treatment. We subsequently selected five developmental stages (0, 6, 12, 24, and 48 h after brassinolide treatment) in *D. zingiberensis* and identified key steps and genes involved in the metabolism of DG and BRs by examining the transcriptome and characteristic metabolite data. Additionally, we identified transcription factors (TFs) and transcriptional regulators (TRs) that modulate metabolite production and accumulation by regulating the transcription of specific pathway gene targets. Finally, we investigated how DG/BR-related genes and key TFs contribute to the balance between DG and BRs through genome-wide methylation. This study provides new insights into the relationship between DG and BRs in *D. zingiberensis* and lays the groundwork for understanding the balance between secondary metabolites and phytohormones.

## Results

### Changes in characteristic metabolites between brassinolide and brassinazole treatments

We extracted several characteristic metabolites from *D. zingiberensis* samples at various times after BL and BRZ (inhibitor of BR synthesis) treatment to better understand the relationship between DG and BRs. A preliminary study was conducted on the effects of BR and BRZ concentrations on DG level. Four concentrations, M1 (0.5 μM), M2 (1 μM), M3 (2.5 μM), and M4 (5 μM), were selected based on a review of the existing literature [15, 35] to observe the changes in DG. The results indicated that the DG levels in the BLM1 and BLM2 treatments followed the same trend, while the BLM4 treatment showed significantly lower levels of DG than the other three groups. Similarly, it was found that the DG content in BRZM1, BRZM2, and BRZM3 treatments followed the same trend, but after BRZM3 treatment DG was significantly lower than in BRZM1 and BRZM2 treatments. DG after BRZM4 treatment was significantly different from the other three groups (Supplementary Data Fig. S1). To ensure specific physiological functions of BL and BRZ rather than toxic effects, BLM2 and BZRM2 were chosen for subsequent experiments.

**Figure 2:**
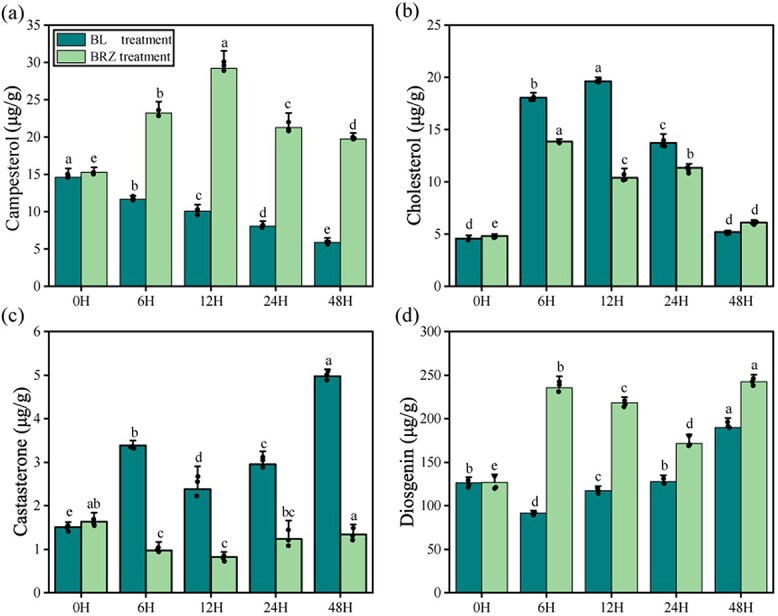
Column chart showing changes in characteristic metabolites after BLM2/BRZM2 treatment. **a** Changes in campesterol content. **b** Changes in cholesterol content. **c** Changes in castasterone content. **d** Changes in DG content. All data are presented as the mean ± standard deviation of three biological replicates, with every data point representing one piece of information. Different lowercase letters represent significant differences between treatments (ANOVA with least-significant difference; *P* < 0.05)

In the BR metabolic pathway, campesterol is an intermediate product. Castasterone is a major active BR [27] and is also used as a precursor substance for BL biosynthesis. We found that campesterol levels continued to decrease by 59.91% at 48 h (Fig. 2a) after BLM2 treatment. Campesterol tended to increase by 91.73% at 12 h (Fig. 2c) and by 29.53% at 48 h after BRZM2 treatment. The amount of castasterone increased by 58.47% at 12 h and 230.94% at 48 h after BLM2 treatment. The castasterone content decreased by ~20% at 6 h/12 h and subsequently regressed to baseline levels at 24 h/48 h after BRZM2 treatment.

Cholesterol is an intermediate of the DG metabolic pathway. Cholesterol showed an overall increasing trend and peaked at 12 h after BLM2 treatment, reaching 327.62% (Fig. 2b). Additionally, cholesterol tended to increase and subsequently increased by 114.83% at 12 h, which was 47.12% lower than that in the BLM2 treatment. Notably, the DG content tended to decrease significantly, by 27.72%, at 6 h after BLM2 treatment. The DG content subsequently decreased to the initial level at 12 h/24 h. In contrast, the DG content increased by 85.34% at 6 h after BRZM2 treatment (Fig. 2d). Additionally, DG significantly increased at 12, 24, and 48 h, with a 90.58% (Fig. 2d) peak increase at 48 h.

In summary, the metabolic pathways of DG and BRs exhibited different trends after BL/BRZ treatment. The trend in castasterone content was also different from that in campesterol content after BL/BRZ treatment. In particular, DG and castasterone synthesis may involve a balance between DG and BRs. This, in turn, suggested that BL treatment results in significant changes in functional metabolite synthesis in *D. zingiberensis*. Therefore, we further investigated the DG and BR pathways after BL treatment via transcriptome sequencing.

### Divergent expression genes in *D. zingiberensis*

To further explore the relationships between DG and BRs in *D. zingiberensis* and the alterations in their gene expression, we carried out a comprehensive transcriptome analysis on *D. zingiberensis* leaves treated with BL for 0, 6, 12, 24, or 48 h. At each time point three samples were collected, each containing >6 Gb of clean bases, and a total of 15 RNA-seq libraries were constructed (Supplementary Data Table S1). Applying the parameters of *P*_adj_ <0.05 and |log_2_-fold change| ≥ 1, we identified 1916, 2005, 1966, and 1839 differentially expressed genes (DEGs) in DZB6H (BR-treated *D. zingiberensis, DBZ*), DZB12H, DZB24H, and DZB48H, respectively, compared with DZB0H. Additionally, we found 791, 783, 798, and 914 upregulated genes and 1125, 1222, 1168, and 925 downregulated genes across these groups (Supplementary Data Fig. S2).

We utilized the Kyoto Encyclopedia of Genes and Genomes (KEGG) database to assign DEGs to metabolic pathways. The analysis revealed that the DEGs were primarily associated with the ribosome, metabolism, flavonoid biosynthesis, biosynthesis of other secondary metabolites, and cytochrome P450 pathways (Supplementary Data Fig. S3). Gene Ontology (GO) term enrichment analysis was used to categorize the majority of the DEGs into various metabolic or biosynthetic processes following BL treatment (Supplementary Data Fig. S3). This increase in downstream metabolites was particularly dominant in DZB24H. In conclusion, KEGG and GO pathway enrichment analyses revealed that the identified DEGs included a proportion of genes related to the plant stress response and had significant effects on protease synthesis, metabolic pathways, and secondary metabolites after BL treatment.

### Weighted gene co-expression network analysis of selected genes

**Figure 3:**
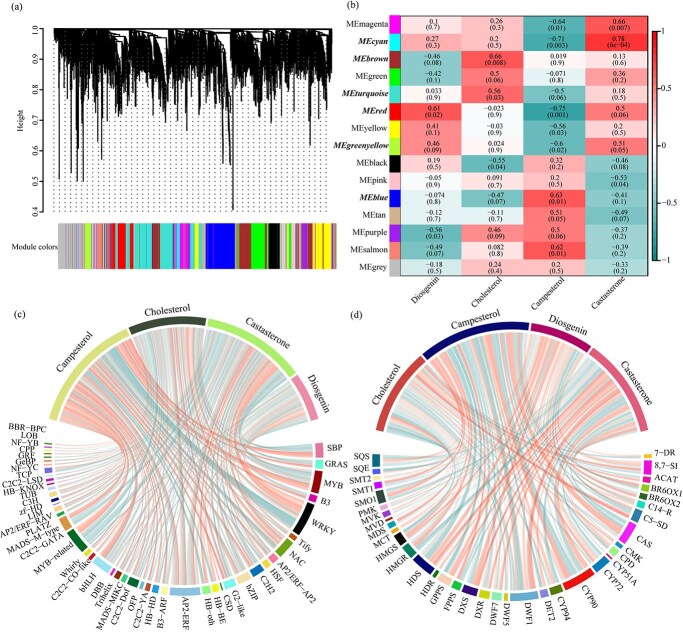
WGCNA of selected genes after BLM2 treatment. **a** Hierarchical clustering dendrogram of gene co-expression modules. Branches marked the 15 modules. **b** Module–phenotype correlation analysis. Each cell in the heat map represents the weighted correlation value and *P*-value between the module and the shape. Each row corresponds to a module and each column corresponds to a metabolite phenotype. Modules significantly correlated with metabolite phenotypes are marked in MEred. Hub genes correlated with important traits are marked as bold and italicized; MEbrown and MEturquoise modules were significantly correlated to cholesterol, the MEblue module was significantly correlated to campesterol, the MEred module was significantly correlated to DG and castasterone, and the MEcyan and MEgreenyellow modules were significantly correlated to castasterone. **c** Correlation analysis of TFs screened in different modules with characteristic metabolites. **d** Correlation analysis of DG/BR-related genes screened in different modules with characteristic metabolites. Red lines represents a positive correlation and blue lines represent a negative correlation.

To probe the relationship between DG and BR-related synthetic pathways in *D. zingiberensis*, we applied weighted gene co-expression network analysis (WGCNA) to the DEG data. WGCNA identified 15 gene modules (Fig. 3a), with gene numbers in each module ranging from 54 to 856 (Supplementary Data Table S2).

Moreover, we identified the red module as critical for DG and castasterone synthesis (Fig. 3b). The MEbrown and MEturquoise modules were significantly related to cholesterol and included genes and TFs related to *8,7-SI*, *C5-SD*, *C-14R*, *DWF1-1/2*, *AP2*s, *C2C2*s, and others. In contrast to castasterone, MEblue is an important component of campesterol and includes *CAS*, *DWF1-3/4*, *DWF5*, *DWF7*, and *SMT1* as well as a large number of TFs, such as *bHLH*s, *bZIP*s, *MYB*s, and *WRKY*s. The MEcyan module, which is enriched in the castasterone synthesis pathway, included *DET2*, *CYP90*, and other related synthesis genes as well as a large number of *WRKY*s. These results indicate that the genes highly related to DG synthesis, which include *CYP72*s, *CYP94*s and *CYP90*s, are mainly concentrated in the MEred module, while the genes related to cholesterol and campesterol show opposite trends, with high relevance in the MEbrown and MEblue modules. The genes of major relevance in the castasterone pathway are concentrated in the MEgreenyellow module. Despite campesterol and castasterone being products of the same pathway, they are composed of genes from completely different modules. In addition, we also found that DG and castasterone, which are downstream products of the steroidal saponin pathway, exhibited the same trend in these modules, indicating crosstalk between the two in the metabolic pathway.

In addition, we performed correlation analyses of TFs/genes and metabolites that screened 46 TFs for a total of 255 copies (Fig. 3c) and 35 DG/BR-related genes for a total of 65 copies (Fig. 3d) in the WGCNA. We found that the BR pathway was regulated by a large number of TFs, whereas the DG pathway was regulated by relatively few TFs. We found that campesterol was most strongly regulated by TFs, most of which were upregulated, and DG was least regulated by transcription factors. Additionally, we found that metabolites that are most closely related to upstream pathways, such as campesterol and cholesterol, were more highly regulated by TFs than their downstream metabolites, namely castasterone and DG.

### Changes in diosgenin/brassinosteroid-related genes in *D. zingiberensis* after brassinolide M2 treatment

**Figure 4:**
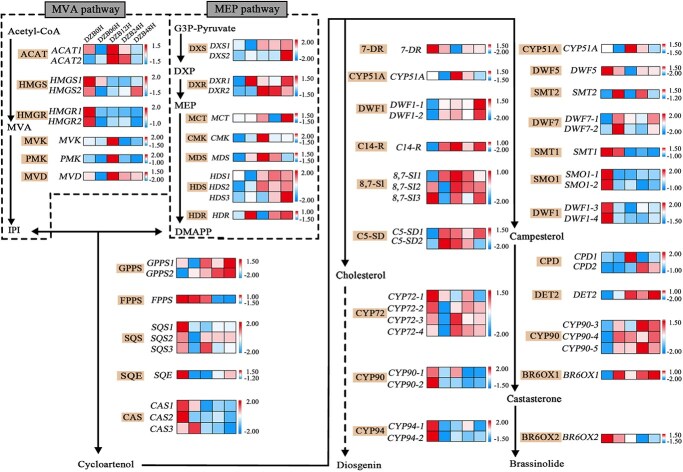
Expression flow chart analysis of several genes related to the DG and BR pathways in *D. zingiberensis* after BLM2 treatment. The dashed line indicates the putative region. Abbreviations: mevalonate (MVA), 2-C-methyl-d-erythritol-4-phosphate (MEP), isopentenyl-5- diphosphate (IPI), 1-deoxy-d-xylulose-5-phosphate (DXP), dimethylallyl diphosphate (DMAPP), C-14 reductase (C14-R), sterol 8,7 isomerase (8,7-SI), sterol 5(6) desaturase (C5-SD), 7-dehydrocholesterol reductase (7-DR), sterol C-24 methyltransferase (SMT), C-4 sterol methyl oxidase (SMO), constitutive photomorphogenesis and dwarf (CPD), deetiolated (DET), and brassinosteroid-6-oxidase (BR6OX).

With respect to the WGCNA and *D. zingiberensis* genome annotation files, we manually curated genes involved in the DG and BR pathways. These included genes in the MVA and MEP pathways. We analyzed the changes in the expression of these genes from WGCNA under BL treatment (Fig. 4). In contrast to that in the MVA pathway, the expression of genes in the MEP pathway increased. Additionally, the expression of *C14-R*, *8,7-SI* and *C5-SD* in the cycloartenol-to-cholesterol pathway was significantly elevated. Among these genes, *DWF5* (*7-DR*) and *CYP51A* are shared by the cholesterol/campesterol pathway. The expression of genes in the cholesterol-to-DG pathway, such as *CYP90-1/2* and *CYP94*s, was also relatively reduced before treatment. In contrast, many genes in the cycloartenol-to-campesterol pathway, such as *SMT1*, *SMO1s* and *DWF1s*, were downregulated. However, the expression of certain genes, such as *DET2*, *CYP90-3/4/5* and *BR6OX1*, significantly increased, leading to the accumulation of castasterone.

Notably, we screened the extent to which the gene isoforms contributed to various metabolites via various WGCNA modules. For instance, we conducted screenings for *DWF1-1/2* in the brown module and *DWF1-3/4* in the blue module. Based on the changes in metabolites, we posit that *DWF1-1/2* play a more significant role in cholesterol production, whereas *DWF1-3/4* have a greater impact on campesterol levels. RNA-seq data were further validated by qRT–PCR. We also validated the BRZ-treated samples by qRT–PCR (Supplementary Data Fig. S4). These data suggest that gene expression in these metabolic pathways is closely tied to downstream metabolite activity. Identifying these significantly differentiated pathway-related candidate genes can also help us understand the molecular mechanisms underlying biosynthesis in *D. zingiberensis*.

### Transcription factor analysis after brassinolide M2 treatment

**Figure 5:**
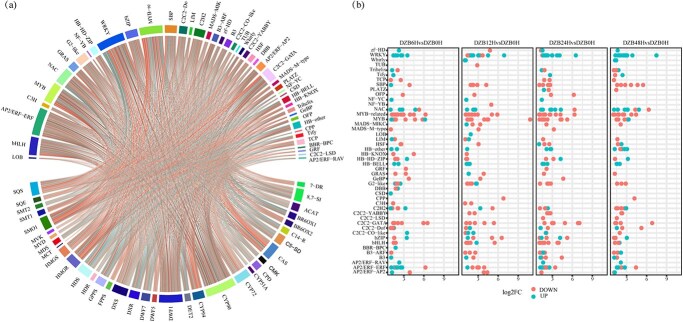
Screening of TFs and DEGs of TFs. **a** Correlation analysis of the TFs with DG/BR-related genes. **b** Fold change maps of DZB6H, DZB12H, DZB24H, and DZB48H differentially expressed TFs. Each TF is represented by a dot. The red and green dots indicate downwards and upwards regulation, respectively.

By analyzing the DEGs at different stages, we identified TFs that constitute an important subset of the DEGs. As evidenced in the literature [36, 37], many changes in metabolites are regulated by TFs. To identify additional TFs linked to DG/BR metabolism, we examined the correlation between 46 TFs associated with metabolites and 35 DG/BR-related genes (Fig. 5a). By combining and screening the metabolite–TF and DG/BR-related genes–TF association analyses, we identified a total of 255 copies. We analyzed the differential modifications of these TFs (Fig. 5b) over time following BL treatment. We identified 118, 111, 138, and 118 TF DEGs in DZB6H, DZB12H, DZB24H, and DZB48H, respectively (Supplementary Data Table S3). Most of these TFs belong to the *WRKY*, *MYB*, *MYB-related*, *NAC*, *C2H2*, *AP2/ERF*, and *HB* families. Importantly, there are certain families in which several different members are regulated concurrently. For instance, the expression of most *WRKY*s was consistently increased, the expression of most *MYB-related*s was consistently reduced, and the expression of other TFs was both reduced and increased. This may suggest that members of different TFs could be involved in cross-regulation.

By performing association analyses of characteristic metabolites and genes related to DG/BR (Supplementary Data Fig. S5), we screened a selection of TRs. We noted a relatively low number of TRs for potential relevance (Supplementary Data Table S4). We found that the expression of AUX/IAA was consistently elevated (Supplementary Data Fig. S5), while most of the TRs, such as GANT, showed an upregulated state. In summary, these TF/TR DEGs suggest that BL treatment of *D. zingiberensis* plays an essential role in the regulation of pathway gene expression, which could regulate the levels of DG and BRs.

### *CAS*s and *CYP90*s play essential roles in regulating diosgenin/brassinosteroid-related genes

Interestingly, we screened *CMT*s, *DRM*s and other plant methylation-related genes from the different modules mentioned above. We believe that the changes in the two pathways involving DG and BRs are to some extent caused by gene methylation. Therefore, we performed whole-genome bisulfite sequencing (WGBS) to probe the specific mechanisms of transcriptional and metabolic changes.

As mentioned above, we used WGBS to gain insight into the specific mechanisms underlying the balance in the DG/BR pathway in *D. zingiberensis*. The overall methylation data are shown in Supplementary Data Fig. S6. We generated 17.74–20.07 Gb of whole-genome methylome data from the above same samples, covering >99% of all cytosine nucleotides (Supplementary Data Table S5). Hyper-methylated differentially methylated regions (DMRs) predominated over hypo-methylated DMRs (Supplementary Data Fig. S7), with DMRs being comparatively elevated in introns and promoters. We also performed KEGG and GO analyses of the DMRs (Supplementary Data Fig. S8), which showed that most DMRs were enriched in metabolic pathways.

**Figure 6:**
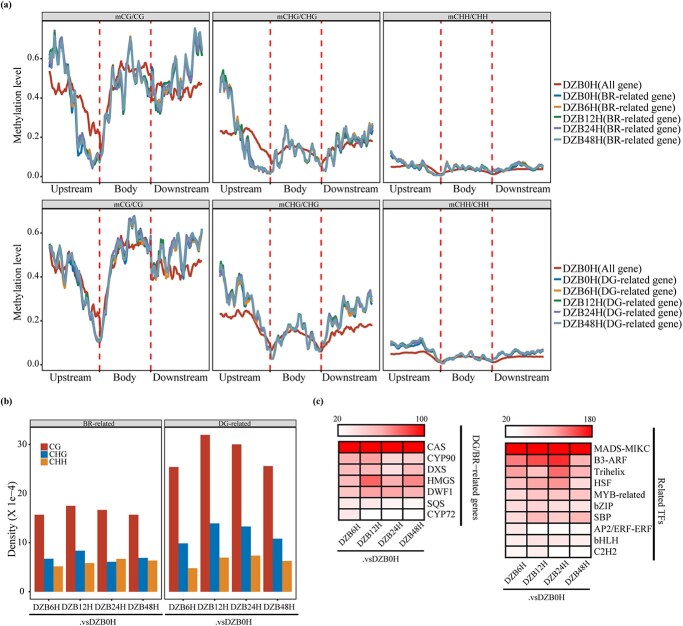
Analysis of differential DNA methylation levels of DG/BR-related genes in *D. zingiberensis* after BLM2 treatment*.*  **a** Methylation status of DG-related genes and BR-related genes in the CG, CHG, and CHH backgrounds. The methylation levels of all genes in DZB0H, DZB0H, DZB6H, DZB12H, DZB24H and DZB48H were determined. **b** Densities of DMCs for DG/BR-related genes in the CG, CHG, and CHH backgrounds under DZB6H, DZB12H, DZB24H, and DZB48H versus DZB0H. **c** CG-DMC heat map of the top-ranked DG/BR-related genes and TFs after BL treatment. The color scale represents the number of genes, restricted to a maximum of 100/180.

We also found that the average methylation levels of the BR-related genes and DG-related genes were significantly different from those of all the coding genes (Fig. 6a). In the CG and CHG sequencing environments, the peaks of upstream methylation levels of BR-related genes appeared alternately, and in the CHH sequence environment the methylation level of BR-related genes was almost always higher than the methylation levels of all genes. However, the methylation levels of the DG-related genes in the CG, CHG, and CHH sequence environments were mostly higher than the overall gene methylation levels.

Unexpectedly, we did not observe an overall change in methylation levels between the DZB0H and BL treatments, suggesting the presence of small amounts of differentially methylated cytosines (DMCs). The density of CG methylation in DG/BR-related genes was much higher than that of CHG and CHH methylation, peaking at DZB12H (Fig. 6b). This indicates that CG methylation plays a major role in the DG and BR pathways. After BL treatment, CG DMCs (Fig. 6c) were particularly prevalent in *CAS*, *CYP90*, *DXS*, *MADS-MIKC*, *B3-ARF*, *Trihelix*, and *HSF*. Furthermore, CG methylation continuously changed.

**Figure 7:**
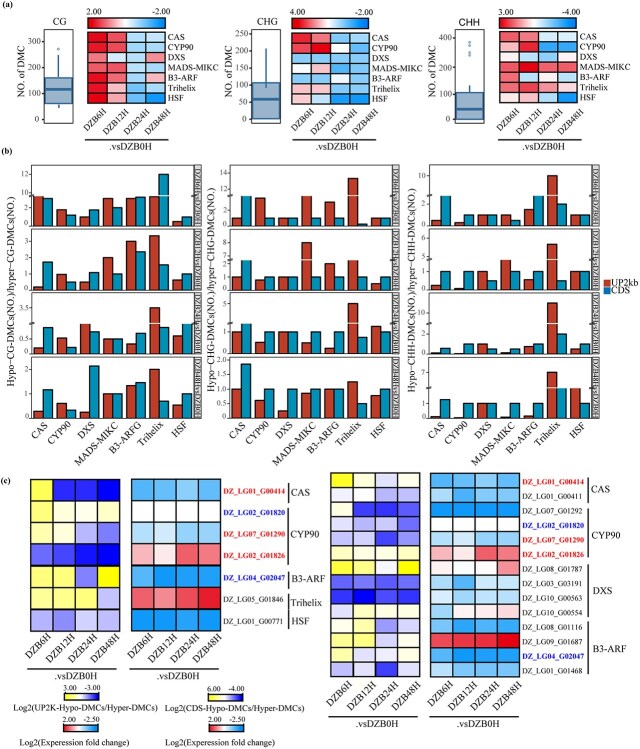
Inverse correlation between CG methylation levels and gene expression. **a** Relative abundance of hyper-methylated and hypo-methylated cytosines in *CAS*, *CYP90*, *DXS*, *MADS-MIKC*, *B3-ARF*, *Trihelix*, and *HSF* after BL treatment in the CG, CHG, and CHH backgrounds. The color scale represents (hypo-DMCs/hyper-DMCs). **b** Relative abundance of hypo-DMCs and hyper-DMCs in the three (CG, CHG, and CHH) backgrounds of UP2kb and the CDS regions of *CAS*, *CYP90*, *DXS*, *MADS-MIKC*, *B3-ARF*, *Trihelix*, and *HSF* after BL treatment. **c** Identification of genes showing an inverse correlation between CG methylation levels and gene expression after BL treatment.

We further investigated the relative amounts of hypo-DMCs and hyper-DMCs in the DG/BR-related gene families and associated TFs in three (CG, CHG, CHH) backgrounds. Among the top seven gene families ranked in our study (Fig. 7a), *CAS* and *CYP90* exhibited relatively high CG methylation. Additionally, in the BL treatment, hypo-DMCs dominated the CDS region of *CAS* in all three sequence environments (Fig. 7b), while hypo-CG-DMCs were dominant in *CYP90* under the UP2kb sequence.

Moreover, we identified 17 genes from the *CAS*, *CYP90*, *DXS*, *B3-ARF*, *Trihelix*, and *HSF* families that exhibited a negative correlation between CG methylation and gene expression in the BL treatment (Fig. 7c). A negative correlation was found in both the upstream and coding regions of the *CAS* (*DZ_LG01_G00414*) and *CYP90* (*DZ_LG07_G1290* and *DZ_LG02_G1826*) genes (Fig. 6c), indicating a possible link between epigenetic modifications and transcriptional changes.

Moreover, there was a higher occurrence of hypo-CG DMCs upstream of B3-ARF (*DZ_LG04_G02047*) (Fig. 7c), and hyper-DMCs were found to be prevalent in the CDS region, resulting in reduced gene expression. This suggests a close association between transcriptional changes and CDS region methylation. *CYP90* (*DZ_LG02_G01820*, labeled blue) exhibited a relative predominance of hyper-CG DMCs in the CDS region, resulting in the non-expression of this gene.

In conclusion, our findings indicate that the *CAS* and *CYP90* gene families could exhibit hypomethylation in *D. zingiberensis*. These findings suggested that epigenetic modifications within these gene families may affect the synthesis of BRs and DG, potentially disrupting the balance between BRs and DG in *D. zingiberensis*.

## Discussion

The synthesis of primary and secondary metabolites by plants is a vital aspect of their life cycle. However, the metabolites can undergo changes when the plant is exposed to stress or other external factors. There is a rebalancing of the metabolites in the plant to cope with the current environment. During this period, the functions of plant secondary metabolites and phytohormones are likely to be replaced for a short period of time. In this study, we found that there is a balance between DG and BRs in *D. zingiberensis*. After the BL treatment, the content of DG decreased the most at 6 h (by 27.72%) (Fig. 2). Differently, the content of DG increased by 85.34% at 6 h after BRZ treatment. However, the castasterone content increased by 58.47% at 12 h and decreased by ~20% at 6 h/12 h. Therefore, to further explore the relationship between DG and BRs, we performed transcriptome, DNA methylome, and association analyses, which had not previously been performed for these steroids. Transcriptome data were used to screen a total of 41 genes related to DG and BRs from the DEGs (Fig. 2). However, the molecular mechanisms of the sterol saponin synthesis pathway have been relatively well studied. The *OSC*, *CYP450*, and *UGT* families [38] have been identified as key players in various species. *CYP90B*s, the ancestral BR biosynthetic genes, are suggested to be essential for sterol 16-hydroxylation [25], an intermediate process in the DG synthesis pathway. *S3GT* has been identified as a key enzyme [39] for the glycosylation of DG in *D. zingiberensis*. Three *UGT*s involved in the glycosylation of steroidal saponin elements [40] were validated in *Paris polyphylla*.

DG is currently produced only by *Dioscorea* and *Paris* species under natural conditions. However, the number of plants capable of producing DG is decreasing due to environmental destruction [41–43]. *Dioscorea zingiberensis* is recognized as a valuable resource in the field of medicinal nutrition in China. DG in fenugreek [44] is regulated by *CYP450*s, *UGT*s and TFs after methyl jasmonate treatment. It has been demonstrated that BL treatment of *Pinellia ternata* [45] modulates endogenous phytohormone levels, increases its own BR content, and regulates its growth and metabolism. Notably, the cholesterol content in *D. zingiberensis* was found to be closely related to *C14-R* and *8,7-SI* (Fig. 3), and the campesterol content was related to *SMT1* and *SMO1,* which exhibited different expression profiles. Additionally, several *CYP450s*, such as *CYP72* and *CYP90-1/2*, were identified, suggesting their significant roles in the DG pathway. The expression of *DET2*, *CYP90-3/4* and BR6OX1 is closely related to the content of castasterone. These genes play a crucial role in maintaining the balance between DG and BRs in *D. zingiberensis*. Moreover, the MVA and MEP pathways are strictly delimited within cellular regions. The MVA pathway is localized in the cytoplasm, whereas the MEP pathway is localized in the plasmodesmata that are specific to the plant [46]. In general, the biosynthesis of steroidal compounds correlated with the MVA pathway. We discovered that, following BL treatment, the MEP pathway displayed greater activity. We assumed that this phenomenon was related to the BL treatment.

Additionally, various TFs have been shown to play crucial roles in plant growth, development, metabolic pathways, and stress responses. For instance, ZFs, MYBs, and WRKYs [47] are predicted to be associated with the synthesis of steroid saponin in *Asparagus officinalis* L. Similarly, WRKYs, MYBs, and bHLHs [48] are speculated to be associated with steroid and terpene skeleton biosynthesis in *Trillium govanianum*. Several studies have shown the involvement of WRKY, AP2/ERF, and other TFs [49, 50] in growth and developmental processes mediated by BRs. In our research we found a positive correlation between the expression of certain TFs (HB-other, AP2, and NAC) and cholesterol. Similarly, C2C2, MYB, and bHLH (Fig. 3) were found to positively regulate the association of campesterol. Notably, a positive correlation was also observed between WRKY and castasterone/DG. Furthermore, our study suggested that these TFs, such as AP2, MYB, WRKY, and bHLH, influence the expression of genes involved in the DG/BR pathway to balance the content of DG/BRs and play a key role in regulating their expression in *D. zingiberensis*.

We found some methylation-related genes in our analysis, suggesting that gene methylation might be the fundamental cause of the above gene changes. Methylation is also a key component of mechanistic studies of many medicinal plants and has been confirmed by sequencing the methylome of *Platycodon grandiflorus*. Among others, the *CYP716* and *bAS* genes [51] are hypermethylated, while epigenetic modifications in these two gene families influence the biosynthesis of platycoside. In recent years, systematic methylation analysis has emerged as a novel approach to achieve a more in-depth understanding of complex genomic information. DNA methylation has a fundamental role in regulating gene expression, biological processes, growth, development, and environmental stress responses [30, 33, 52–54]. Furthermore, the methylation of transposable elements situated within or near genes can influence their transcription under both developmental and stress conditions [55–57]. The DNA methylation of kenaf seedlings [58] suggested that genes or TFs such as ARFs, PP2C, and starch synthase may be involved in the regulation of flowering in kenaf seedlings.

In our study, we created the largest methylation resource for *D. zingiberensis* to date and constructed a comprehensive epigenome map (Fig. 6). We identified hypomethylation of DG/BR-related genes and TFs, such as *CAS*s, *CYP90*s, *DXS*s, *MADS-MIKC*s, *B3-ARF*s, *Trihelix*s and *HSF*s, which are likely to play crucial roles in the biosynthesis of DG and BRs in *D. zingiberensis*. Furthermore, *CAS*s and *CYP90*s (Fig. 7) play important roles in maintaining the balance between BRs and DG in *D. zingiberensis*. BL treatment causes an increase in the methylation levels of certain genes, including some *CYP90*s, which leads to a temporary decrease in DG content. Our characteristic metabolites analysis supported these findings. In addition, not only do DG/BR-related genes have a significant impact on this equilibrium process, but numerous other crucial TFs, such as *MADS-MIKC*s, *B3-ARF*s, *Trihelix*s, and *HSF*s, have also been identified as key players in this process. Notably, DNA methylation is known to influence plant growth and development, and our findings suggest that this process might also play an important role in the regulation of growth. These studies provide a valuable foundation for future investigations into the methylation characteristics of *D. zingiberensis* and the regulation of gene expression in the DG and BR pathways.

In conclusion, our research reveals that BL treatment directly affects the level of gene methylation in *D. zingiberensis*, which in turn impacts the expression of genes or TFs involved in the DG and BR pathways, leading to alterations in related metabolism. We present a comprehensive analysis of the DG and BR pathways, illustrating that the two pathways share certain genes and TFs but exhibit differences. This study not only helps define the regulatory network of characteristic metabolites in *D. zingiberensis* but also paves the way for understanding the balance between DG and BRs in plants. This study provides novel insights to add to the currently scarce molecular research results on *D. zingiberensis*, offers a promising new avenue for determining the balance between secondary metabolite and phytohormone regulation and lays the groundwork for further research into the associated mechanisms.

## Materials and methods

### Plant materials and treatments

In this study, mature seeds of *D. zingiberensis* were collected from Shiyan City, Hubei Province, and used as research material. The seeds were sown and maintained in a conservatory at Wuhan University at a temperature of 26°C with a 16-h light/8-h dark photoperiod. Ten months later, uniformly grown and healthy *D. zingiberensis* leaves were chosen for treatment. Each plant was individually sprayed with equal volumes of different concentrations [M1 (0.5 μM), M2 (1 μM), M3 (2.5 μM), and M4 (5 μM)] of BR or BRZ. Leaves taken 0 h (DZB0H, DZZ0H), 6 h (DZB6H, DZZ6H), 12 h (DZB12H, DZZ12H), 24 h (DZB24H, DZZ24H), or 48 h (DZB48H, DZZ48H) after spray treatment were immediately frozen in liquid nitrogen. Each time point was represented by three replicates. For further experiments, all samples were stored in a −80°C freezer.

### Quantification and analysis of characteristic metabolites

The extraction and quantification of phytosterols by GC–MS were performed as follows. Five leaf samples (each with three replicates) were crushed in liquid nitrogen. To 50 mg of powder, 2 ml of a chloroform:methanol (2:1) solvent was added, and the mixture was incubated for 1 h at 75°C in a water bath. Then, the mixture underwent incubation at 90°C for 1 h after the addition of 500 μl of 6% KOH methanol solution. The solution was passed through 0.22-μm membrane solution filters. The extracted solution was transferred to a new tube and vacuum-dried at room temperature after adding 500 μl of water and 500 μl of hexane three times and shaking for 30 s each time. For derivatization, 50 μl of *N*-methyl-*N*-(trimethylsilyl) trifluoroacetamide was added to the dried sample at room temperature for 30 min. Next, to determine the phytosterols, 150 μl of hexane was added. A Thermo Trace GC gas chromatograph, equipped with a TG-5 MS column, was used to analyze phytosterols [59–61]. The separation process began with an initial temperature of 80°C for 1 min, followed by heating at a rate of 15°C per minute until it reached 290°C, and then maintaining this temperature for 10 min. The ion detection range spanned from *m*/*z* = 50 to 650.

For the extraction and quantification of DG, 50 mg of ground powder was mixed with pre-cooled isopropanol (1 ml) and shaken for 20 min at 200 rpm in a 40°C incubator. The mixture was sonicated for 30 min and then centrifuged at 12 000 rpm for 10 min, and passed through 0.22-μm membrane solution filters. The elution process involved the use of two mobile phases: A was 5 mM NaAc in water and B was 5 mM NaAc in methanol. The process comprised the following steps: 0–2 min, 65% B; 2–10 min, 65–95% B; 10.5 min, 95–65% B; and 10.5–12 min, 65% B. DG ions (*m*/*z* = 415.3202) were monitored using Target-SIM in positive ion mode.

The extraction and quantification of castasterone were conducted as follows. One hundred milligrams of the above samples was ground in liquid nitrogen, added to 1170 μl of an 80 acetonitrile:19 water:1 formic acid solution (v/v), vortexed for 1 min, sonicated at 4°C for 30 min while being shielded from light, and stored at −20°C. The samples were centrifuged at 12 000 rpm for 20 min at 4°C, and the supernate was filtered under positive pressure into an Ostro 25 mg 96-well desquamated plate. The mixture was then eluted once with 200 μl of the standard product and stored at −80°C. The elution process involved the use of two mobile phases: A was 0.05% formic acid in water and B was 0.05% formic acid in acetonitrile. The correlation gradients were as follows: 0–1 min, 2–10% B; 1–10 min, 10–70% B; 10–11 min, 70–95% B; 11–11.1 min, 95–2% B; and 11.1–13 min, 2% B. Subsequent mass spectrometry was conducted in positive/negative ion mode, and the ion pairs were detected using MRM mode. For more information on the above metabolites, refer to Supplementary Data Table S6.

### RNA sequencing and data analysis

Total RNA was isolated from leaf samples of DZB0H, DZB6H, DZB12H, DZB24H, and DZB48H (with three replicates each) and the NEBNext Ultra RNA Library Prep Kit for Illumina (NEB, USA) was used to construct sequencing libraries. Eligible libraries were sequenced on the Illumina Novogene platform based on their effective concentration and the required data volume.

The raw files of the fluorescence images generated by the Illumina platform were converted into short reads by means of base calling. These short reads were stored in the FASTQ format [62, 63]. The clean reads were in alignment with the *D. zingiberensis* reference genome, followed by analysis using DESeq2 (version 1.36.0). We applied *P* < 0.05 and minimum fold change |log_2_(fold change)| ≥ 1 to identify significant differences between treated and control samples. Enrichment terms for DEGs in the GO [64, 65] and KEGG [66–68] databases showing significant differences were adjusted using TBtools [69] with a *P* value (FDR) <0.05. The default parameters of TBtools were used for functional annotation and biological pathway analysis of DEGs in the KEGG and GO databases.

### Total RNA extraction and quantitative real-time PCR analysis

For quantitative real-time PCR (qRT–PCR) analysis, all leaf samples (with three replicates each) were analyzed. Total RNA was isolated using TRIzol (TransGen, Beijing). From each sample, 1 μg of RNA was obtained, and a cDNA template was created with cDNA Synthesis SuperMix (TransGen, Beijing). The All-in-One First-Strand cDNA Synthesis SuperMix for qRT–PCR (TransGen, Beijing) was used to perform the method to investigate key genes in different samples and to confirm the reliability of the transcriptome data. Supplementary Data Table S7 lists the primers of the key genes. *DzActin* was utilized as the internal reference gene, and the 2^-∆∆CT^ method was applied.

### Weighted gene co-expression network analysis

Based on the above findings, we incorporated the differential expression data into a WGCNA [70]. This analysis was performed using the WGCNA package in R software. The automatic network construction function was used to obtain co-expression modules with default parameters, except for a soft threshold power. Unsigned networks were detected using the Pearson method, and a topological overlap measure (TOM) was determined for each gene pair. Hierarchical clustering was performed using mean linkage based on the dissimilarity of TOM. Subsequently, a dendrogram was constructed, and the size of the minimum gene module was set. The steroid saponin content of the samples was used as phenotypic data to calculate Pearson correlations between each gene module and the various steroid saponins, as well as between the various treated samples. After identifying the gene modules that were significantly linked with the sterol saponin profile, inter-gene expression correlation coefficients >0.1 (*P* ≤ 1e−6) were chosen as candidate genes.

### Whole-genome bisulfite sequencing and data analysis

High-quality genomic DNA was isolated from the leaf samples using a DNA Plant Kit. Using Covaris S220, 0.1 μg of genomic DNA, along with 0.5 ng of λ DNA as an internal standard, was sheared to 200–300 bp fragments. Samples were then sequenced on the NovaSeq platform (Illumina, CA, USA) to achieve a 30× depth of coverage. Bisulfite-treated reads were aligned to the *D. zingiberensis* genome using Bismark software (version 0.16.3) [71, 72].

The distribution of methylation cytosine sites across *D. zingiberensis* chromosomes and various functional components of the genome was analyzed. A binomial test was used to identify methylated sites using methylation counts, total counts and non-conversion rates (*P*-value <0.05).

DSS software was used to identify DMRs [73–75]. Based on the DMR distribution in the genome, genes associated with DMRs were categorized as having DMR overlap within the region from the transcription start site to the transcription end site or the promoter region. The GOseq R package [64] was used to conduct a GO enrichment analysis of genes associated with DMRs, with correction for gene length bias. KOBAS software [67, 68] was used to perform statistical enrichment testing of DMR-associated genes in KEGG pathways.

### Statistical analysis

We performed the statistical analysis and generated the graphs using the R project (https://www.r-project.org/). We explored the correlation between the steroidal saponin content and gene expression among different samples using Spearman analysis. SPSS was used to perform a one-way ANOVA to analyze the impact of BL/BRZ on metabolic parameters. A least significant difference test was used to calculate the comparisons of mean values (*P* < 0.05).

## Supplementary Material

Web_Material_uhae055

## Data Availability

The RNA-seq raw data described in this paper have been deposited in the National Genomics Data Center, Beijing Institute of Genomics (China National Center for Bioinformation), Chinese Academy of Sciences, under the Genome Sequence Archive (GSA) accession numbers CRA010777 and CRA010789 (https://bigd.big.ac.cn/gsa).
