# Supplementary figures and images for: Genome-wide methylation, transcriptome and characteristic metabolites reveal the balance between diosgenin and brassinosteroids in Dioscorea zingiberensis
Source: Hortic Res. 2024 Feb 23;11(4):uhae056. doi: 10.1093/hr/uhae056 (PMC11040209; doi:10.1093/hr/uhae056)

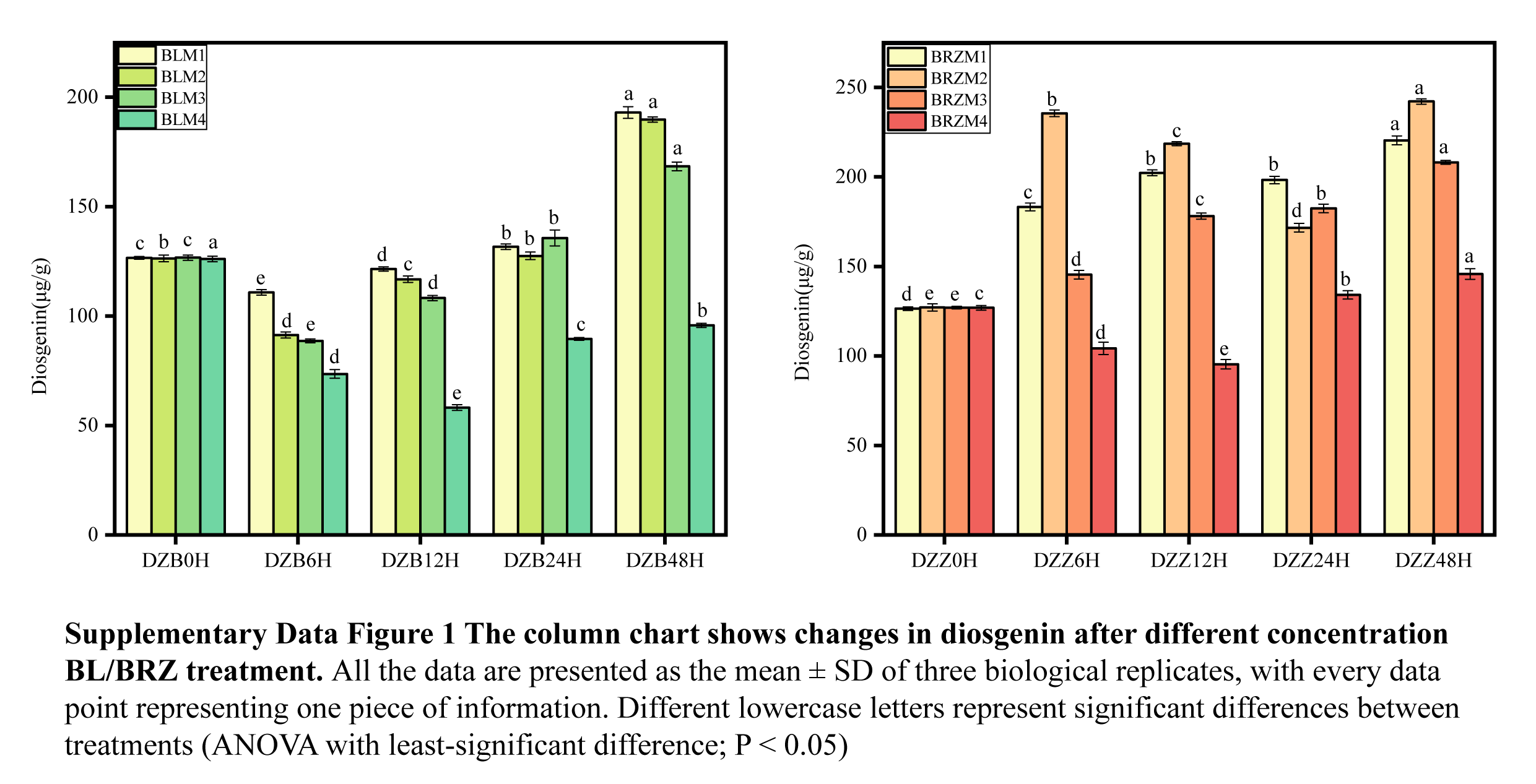

Supplement: Web_Material_uhae055 [file web_material_uhae056.zip › Supplementary Data Figure1.tif]

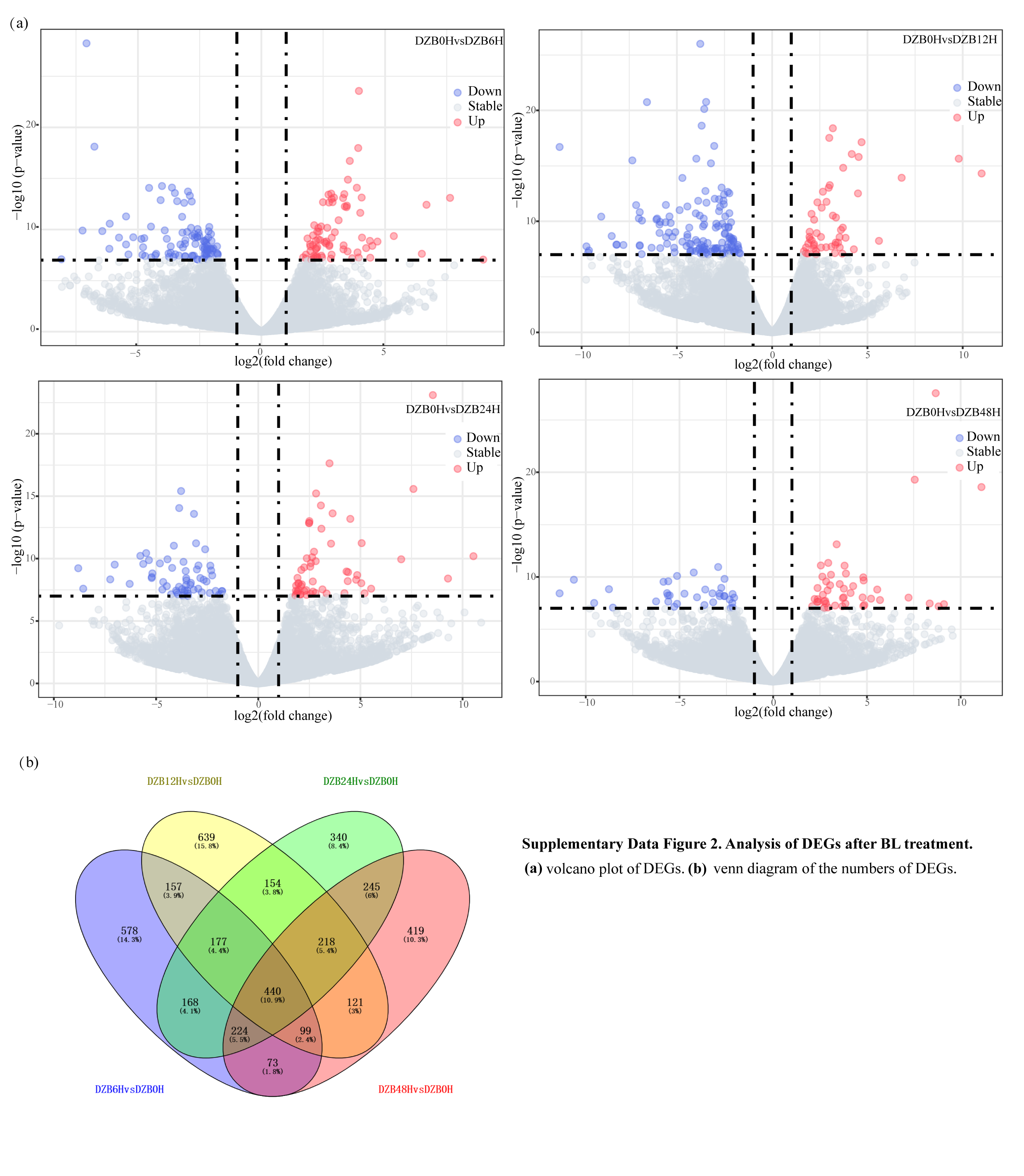

Supplement: Web_Material_uhae055 [file web_material_uhae056.zip › Supplementary Data Figure2.tif]

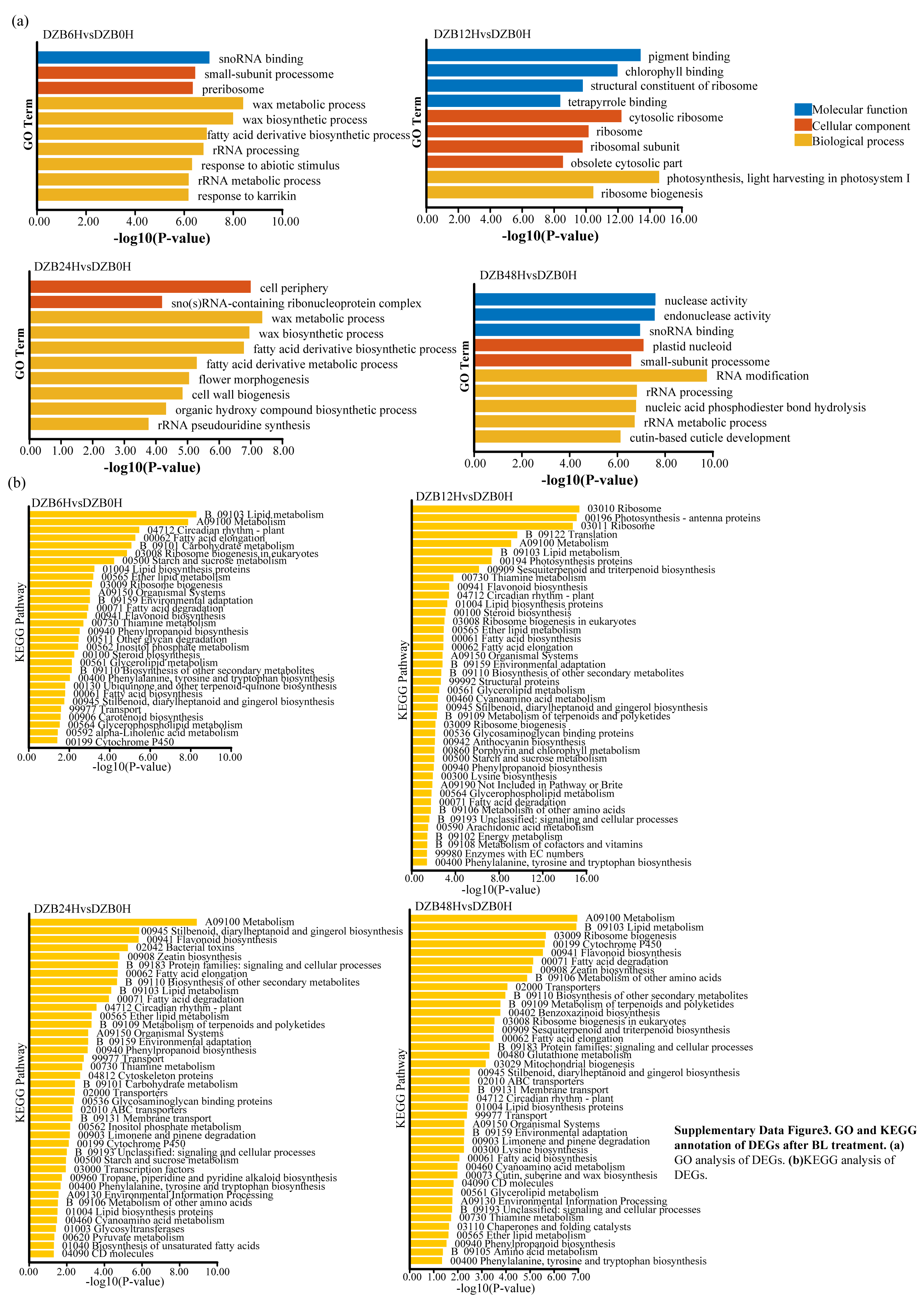

Supplement: Web_Material_uhae055 [file web_material_uhae056.zip › Supplementary Data Figure3.tif]

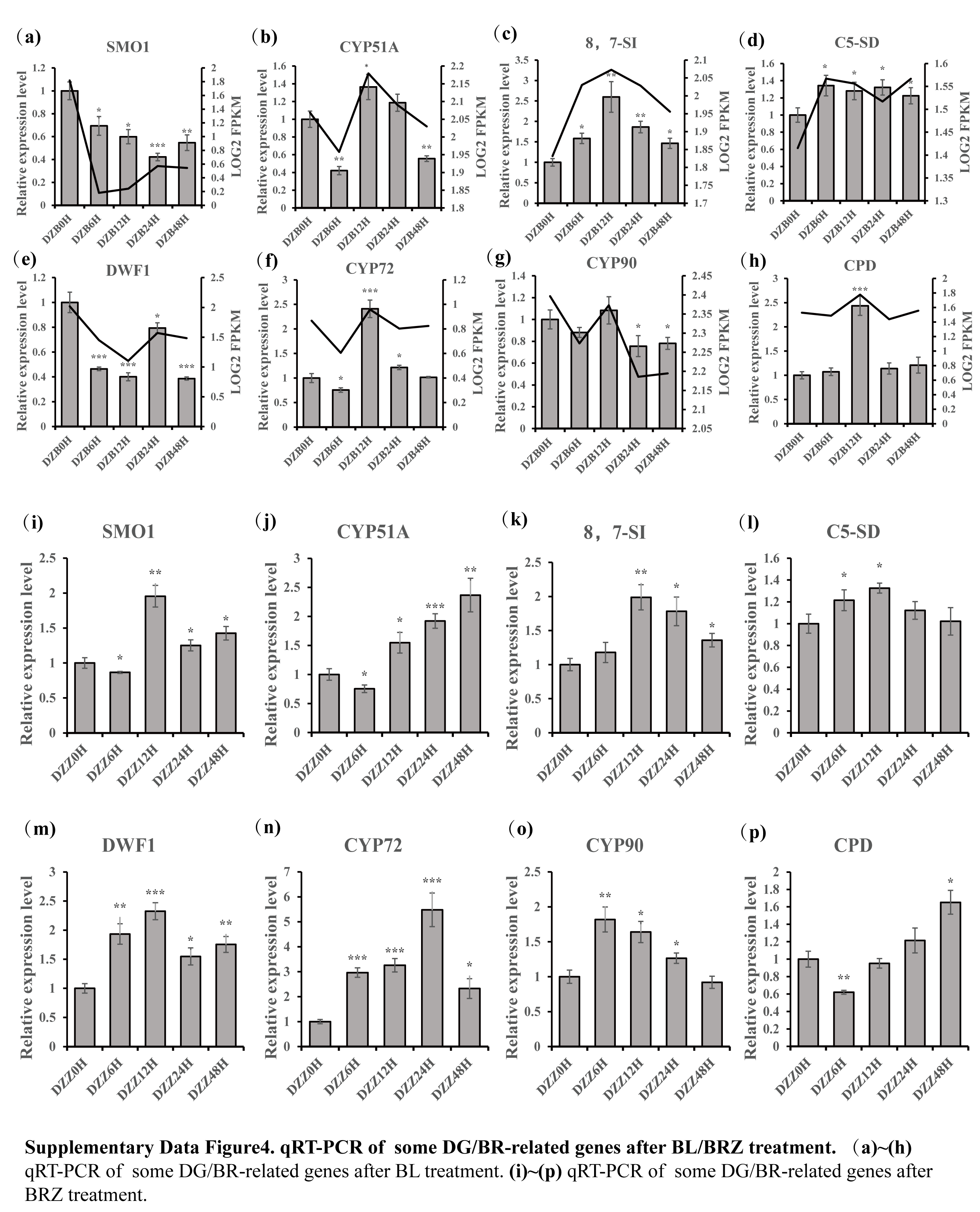

Supplement: Web_Material_uhae055 [file web_material_uhae056.zip › Supplementary Data Figure4.tif]

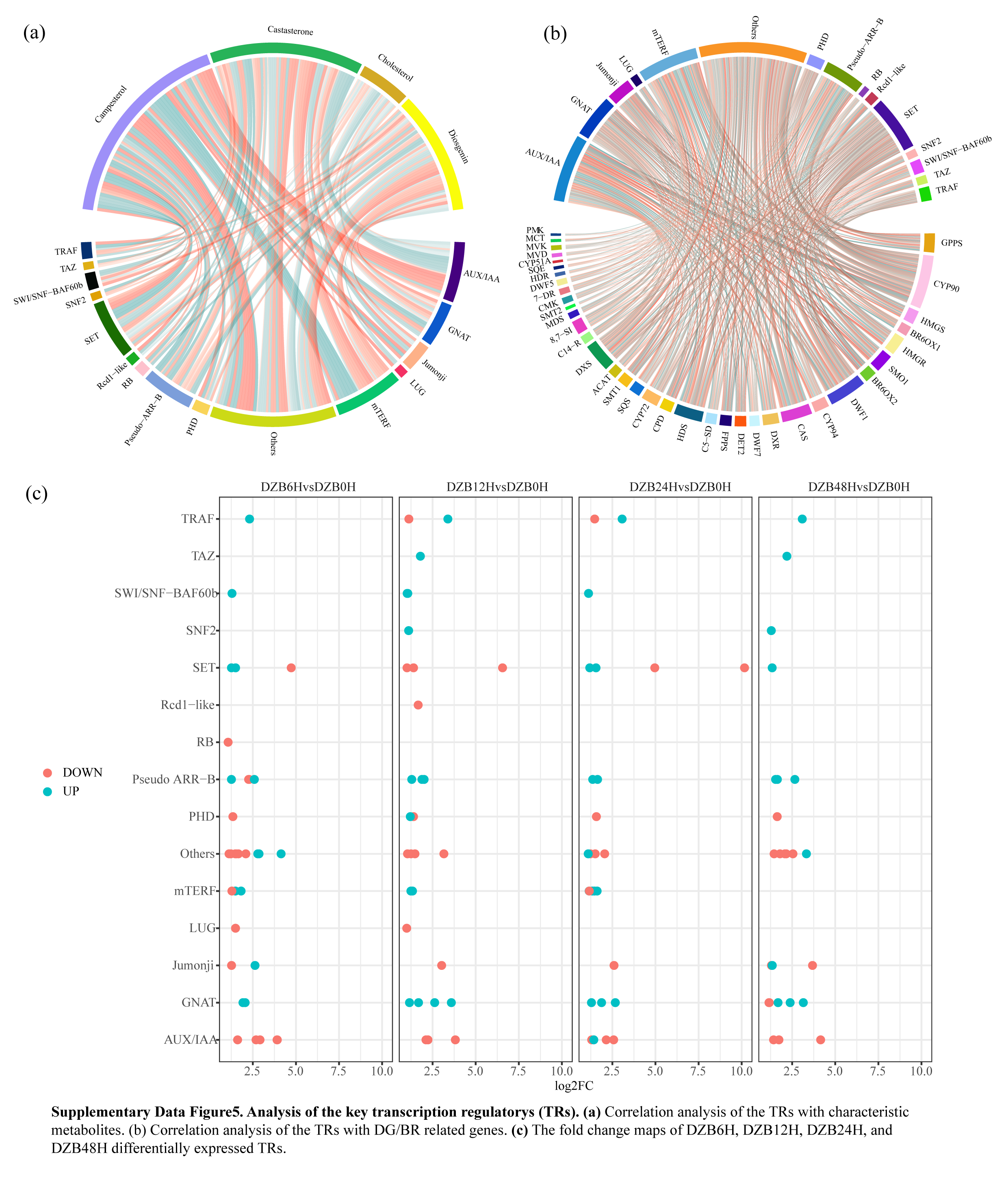

Supplement: Web_Material_uhae055 [file web_material_uhae056.zip › Supplementary Data Figure5.tif]

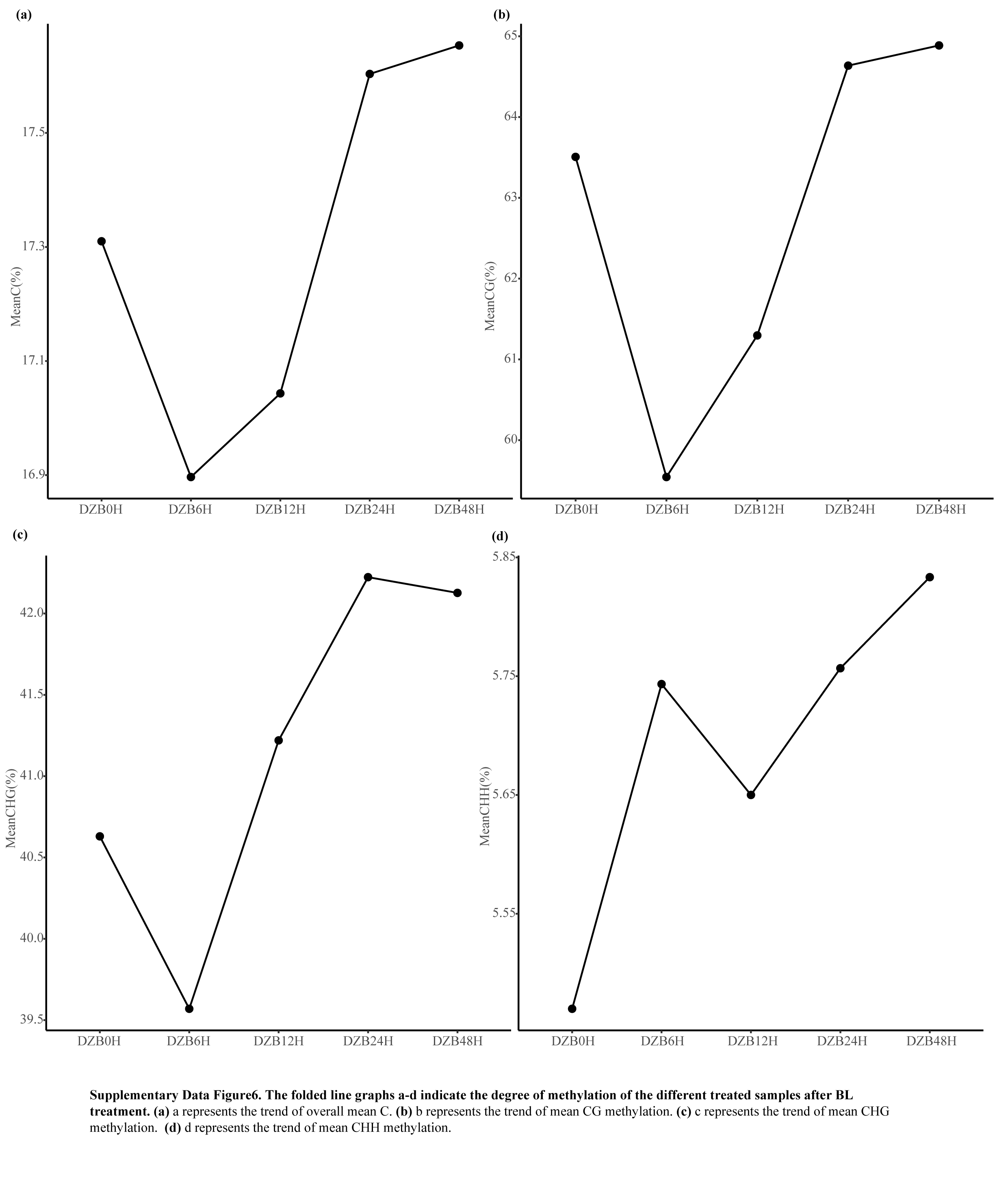

Supplement: Web_Material_uhae055 [file web_material_uhae056.zip › Supplementary Data Figure6.tif]

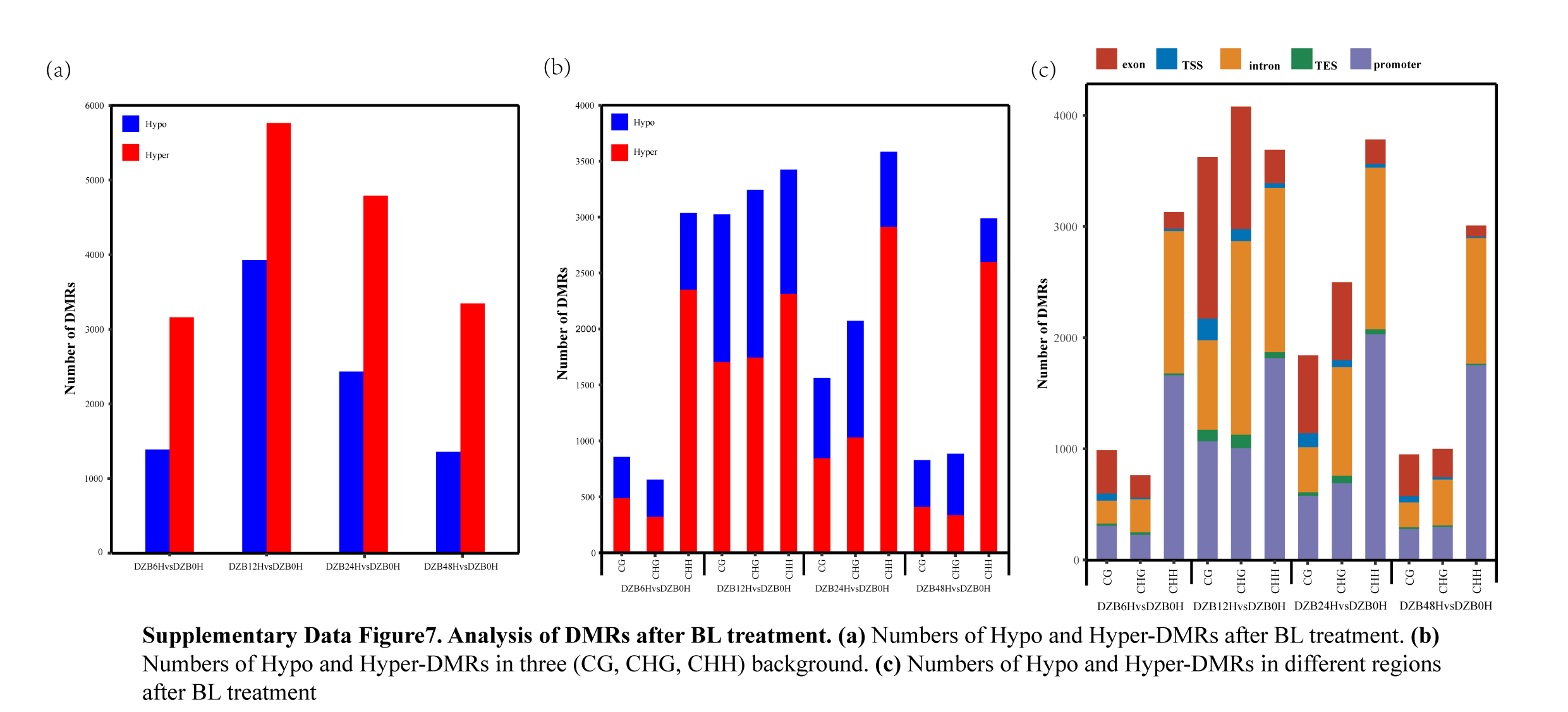

Supplement: Web_Material_uhae055 [file web_material_uhae056.zip › Supplementary Data Figure7.tif]

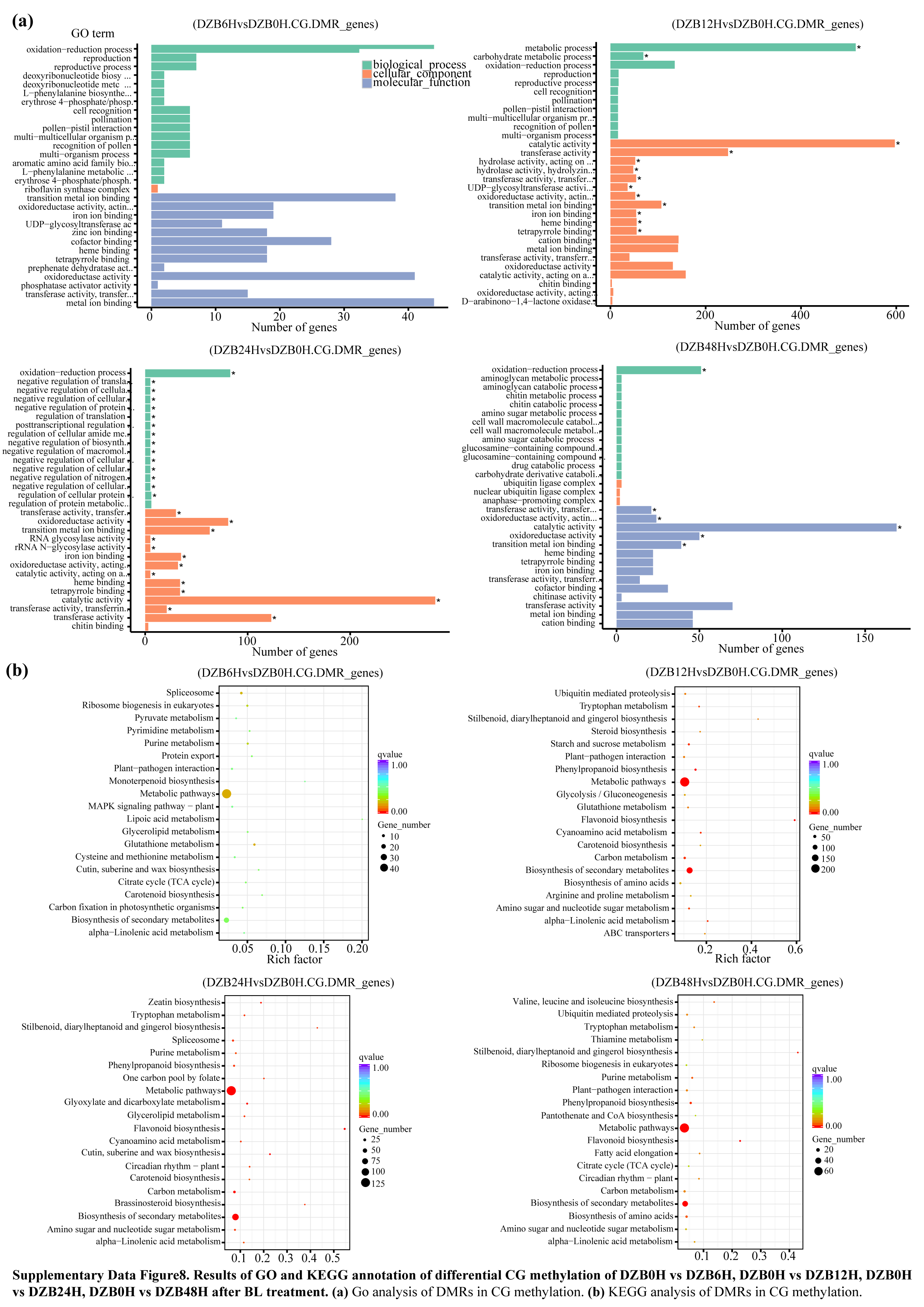

Supplement: Web_Material_uhae055 [file web_material_uhae056.zip › Supplementary Data Figure8.tif]
